# Ventricular fibrillation and Takotsubo cardiomyopathy triggered by media panic on COVID‐19: A case report

**DOI:** 10.1002/ccr3.3423

**Published:** 2020-12-03

**Authors:** Dirk Habedank, Roland Thieme, Angelika Bublak, Felix Heinemann, Sebastian Spencker, Iskandar Atmowihardjo

**Affiliations:** ^1^ DRK Kliniken Berlin Köpenick Medizinische Klinik Kardiologie Berlin Germany

**Keywords:** anxiety disorder, COVID‐19, defibrillator, J‐wave, takotsubo cardiomyopathy, ventricular fibrillation

## Abstract

Takotsubo cardiomyopathy has potentially lethal complications and can be caused by a media‐induced diffuse atmosphere of life threatening and panic in preconditioned patients.

## Graphical abstract

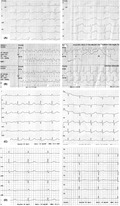

## 1 INTRODUCTION

We report a case of Takotsubo cardiomyopathy (TTC) induced by diffuse fear of the COVID‐19 pandemia and aggravated to ventricular fibrillation. After successful resuscitation, the patient developed temporarily a J‐wave after event. Though potentially life threatening in the acute phase, both J‐wave and TTC are reversible with receding of edema.

Takotsubo cardiomyopathy (TTC) was initially regarded as a rare and self‐limiting event. As awareness among clinicians grew over the last 30 years, the proportion of TTC in acute coronary syndromes was increasingly diagnosed and is currently 1%‐2%.^1^ Moreover, TTC might induce serious rhythm complications as ventricular tachycardia, of which an incidence between 5.3% ^2^ and 8.0% ^3^ is reported. The risk of torsades des pointes is elevated in patients with QT_c_ interval prolongation > 500 ms and of ventricular flutter with augmented J‐waves.^4^ We report a case of TTC that was induced by diffuse fear of the COVID‐19 pandemia, aggravated to ventricular fibrillation (VF), and developed temporarily a J‐wave in the acute phase and after event.

## 2 CASE REPORT

**Figure 1:**
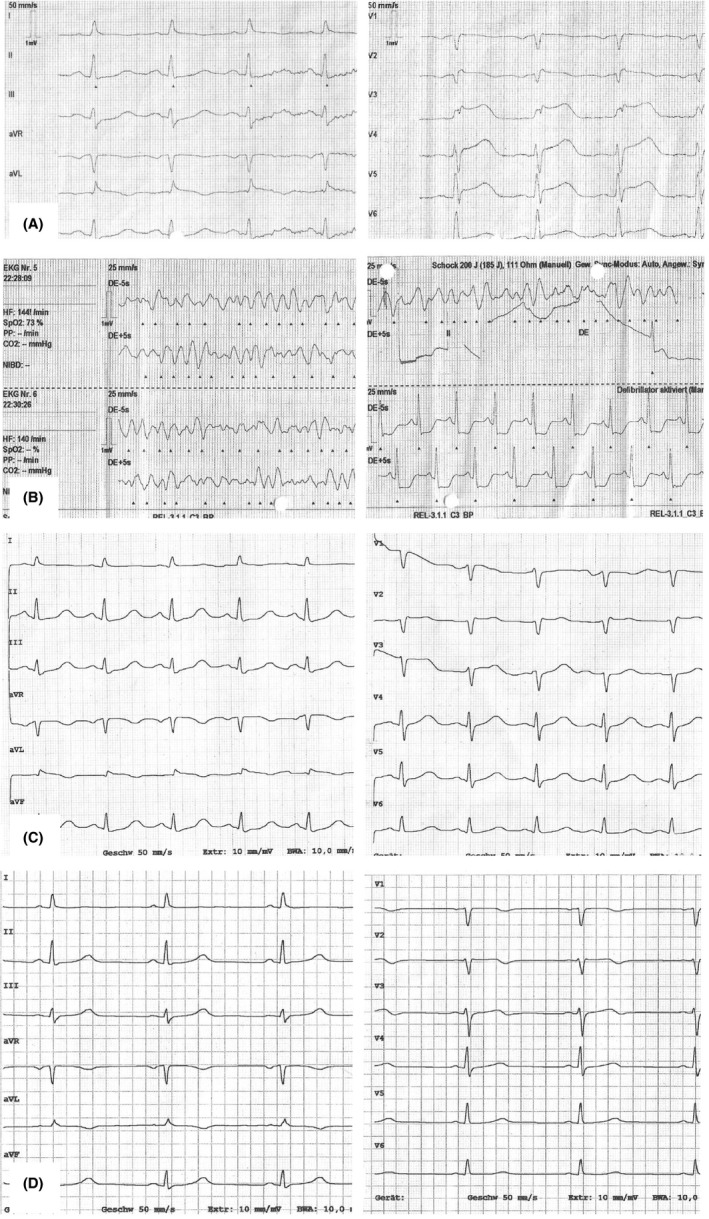
ECG. A, Before ventricular fibrillation (VF) with ST elevation. B, VF and defibrillation. C, 1 h after VF with J‐wave in leads V1 and V2. D, 4 d after VF nearly recovered

**Figure 2:**
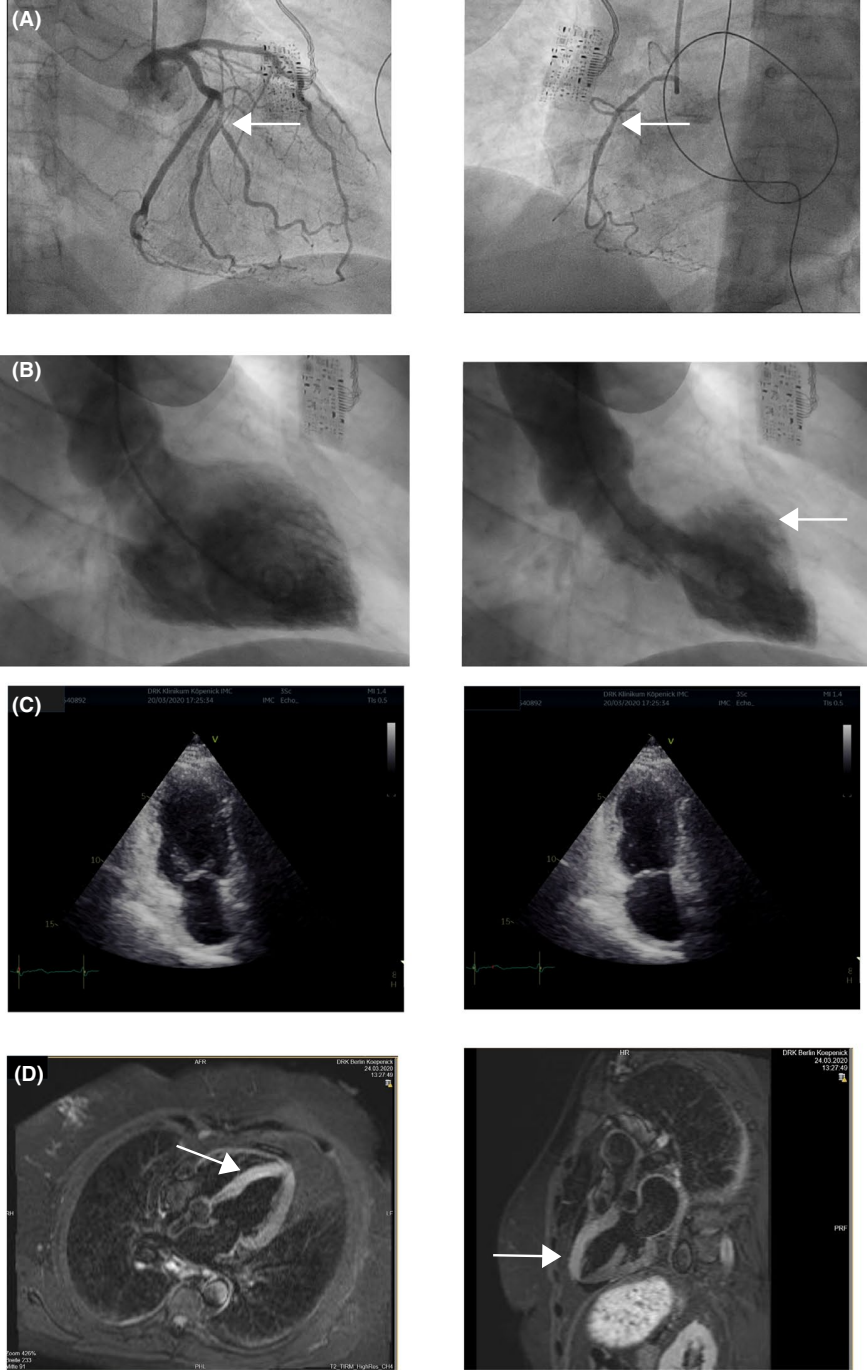
Images. A, Coronary angiogram (arrows: sclerosis in the right coronary and left circumflex artery). B, Ventricular angiogram (arrow: mid‐anterior pronounced akinesia). C, Echocardiography 24 h after VF (left: diastole, right: systole). D, Cardiac MRI (arrow: edema of the anterior wall)

We report on a 63‐year‐old woman who works as official in charge at a governmental service unit. She had a history of anxiety, mild depression, and arterial hypertension. The antidepressant had been withdrawn 2 years ago, and current medication consisted of ACE‐inhibitor only. At the beginning of official shutdown caused by the COVID‐19 outbreak, she had to leave her authority site on March 16th to work in a home‐office constellation. She was very upset by lockdown regulations, the necessity to install a working place at home, and she was listening two whole days to radio and TV reports on emerging news about this pandemia. Throughout March 18th, she felt increasingly stressed by both threatening news and requirements of her employer, culminating in increasing chest pain in the evening of March 18th. As pain worsened, she called the emergency medical service. ECG showed ST elevations 0.4 mV from J‐point in leads V2 to V4, 0.1 mV in lead aVL, and a QT_c_ = 522 ms by Bazett's resp. 477 ms by Fridericia's formula (Figure 1A). The emergency physician administered aspirin 500 mg and heparin 5000 IU iv and informed the nearest catheter laboratory about the immediate arrival of a patient with anterior myocardial infarction. During lying transportation from her flat into the ambulance, symptoms aggravated, and just within ambulance, the monitoring ECG showed a ventricular fibrillation (Figure 1B) and she lost consciousness. The patient was defibrillated with 200 J anterior‐posterior to a stable sinus rhythm and regained spontaneous circulation immediately. The patient was directly transferred into the catheter laboratory of our hospital, and the coronary angiography showed a moderate coronary sclerosis that could not be causative to the ECG changes and the ventricular fibrillation. Contrast filling of the left ventricle proved the diagnosis of a mid‐ventricular pronounced Takotsubo cardiomyopathy (Figure 2A and B).

The patient was admitted to the intensive care unit for 24 hours and to an ECG monitoring ward for another 48 hours. There were no further rhythm disturbances, and the patient remained free of cardiac symptoms. All laboratory changes normalized within 2 days: Troponin T was at maximum 152 ng/mL (upper reference level 50 ng/mL), maximum creatine kinase 120 U/L (reference 167 IU/L), and leukocyte count 21‐9 Gpt/L. ECG showed a J‐wave in V2 and receding ST elevations 1 hour after defibrillation (QT_cB_ = 501 ms, QT_cF_ = 453 ms; Figure 1C) and only sketchy terminal negative T‐waves in V2‐3 after 6 days, but no giant T‐waves as pathognomonic for TTC (QT_cB_ = 420 ms; Figure 1D). Left ventricular ejection fraction was calculated to 35\% at admission and recovered to normal within 4 days, with the echocardiography showing still moderate hypokinesia in the mid‐anterior section (Figure 2C) and cardiac MRI proving significant edema in the entire anterior and septal wall (Figure 2D). Despite the normally benign course of TTC, we decided together with the patient for implantation of an ICD because her history of anxiety and treated depression made a recurrence possible. A subcutaneous ICD was implanted on March 25th and the patient discharged 2 days later.

## 3 DISCUSSION

Our patient represents the typical population at risk for TTC: About 91% of patients are postmenopausal women, the mean age is 67 years,^5^ and—considering the prevalence in older patients—a bystander coronary artery disease as obvious by coronary plaques in our patient may be present.^6^ A history of anxiety (27%),^1^, ^7^ and depression is common, so that negative emotions and natural disasters ^8^ can set the final trigger for development of TTC. Recent data of the large International Takotsubo Registry show that the prevalence of acute psychiatric disorders in elderly patients is significantly lower than in younger patients (14.1% vs 5.6%).^9^ However, both physical (intracranial bleeding or cerebral trauma) and psychical (sudden emotional stress) brain injuries trigger left ventricular ballooning syndromes in a wider sense, hence subsumed under the term “neurogenic cardiomyopathy”.^10^, ^11^ However, the complication of VF is rare.^2^, ^3^ The J‐wave visible within the first 48 hours after event is not expression of a concealed Brugada syndrome, though visible in the Brugada‐typical lead V2, but explained by myocardial edema. Shimizu et al found an augmented J‐wave in 9 of 31 (29%) consecutive patients,^4^ and several case reports support this finding.^12^, ^13^, ^14^ In summary, these ECG alterations and the transient J‐wave in TTC are explained as result of transmural voltage gradient leading to increased dispersion of repolarization among neighboring cells. In detail, epicardial cells own a strong transient outward potassium current (I_to_) that is much weaker in endocardial cells. This I_to_ causes the prominent notch during early repolarization in epicardial cells.^15^ Acute regional myocardial ischemia leads to a heterogeneous loss in I_to_ in epicardial cells that might be augmented by increased ATP‐sensitive K^+^ and decreased Na^+^ and Ca^++^ current. Hence, the action potential is regionally shortened and the heterogeneity between neighboring cells allows a reentry in action potential phase 2 with development of ventricular tachycardia or VF.^16^ In a strict sense, these alterations of action potential have been shown in myocardial ischemia and hereditary disease,^17^ and the explanation of J‐wave in TTC is a transmission of this model. It was adapted to TTC by Shimizu et al, who located the vulnerable zone between the hypercontractile (basal) and the stunned (apical) segments of the left ventricle.^4^ These changes at subcellular level may lead to a prolonged QT_c_ interval as seen in our patient. A prolongation of QT_c_ was detected in 47.7% of 1750 patients ^5^ but in all 9 of 178 patients with polymorphic VT or VF.^18^ Though potentially life threatening in the acute phase, this condition is reversible with receding of edema.

Despite the normally complete recovery, a recurrence of combined TTC with VF is possible.^19^ Moreover, observation of transient J‐wave indicates on high risk of ventricular arrhythmias. Common heart failure therapies did not prevent recurrence of TTC,^20^ although an expert commission voted in favor of beta‐blockers in patients with increased sympathetic tone and anxiety disorders.^6^ The implantation of a permanent ICD is discussed controversially. Current guidelines regard the implantation of ICD as uncertain (evidence level C),^21^ This in line with a recent study that did not note a life‐threatening arrhythmia in 9 TTC patients after device implantation during follow‐up (range 30 to 2920 days).^22^ However, another working group showed a one‐year mortality of 44% in patients after life‐threatening arrhythmias compared with 10% in patients without arrhythmias ^18^ and these authors recommend temporary wearing of a cardioverter‐defibrillator vest until ECG and left ventricular function have recovered.^23^ As psychiatric comorbidity is concerned, a recent study showed that emotion regulation in former TTC patients remains impaired even 27 months after event and processing of emotions is different from normal in frontal, parietal, occipital, and cerebellar brain regions.^24^ Considering these studies and the history of our patient with anxiety disorder, depression, and acute development of J‐wave, we decided to implant a permanent ICD. We should note critically that we did not ask for a psychiatric support, as a treatment for the etiology might help in avoidance of recurrence of such condition. Although trigger events leading to TTC are diverse, the fear of a pandemia is new as causative agent for TTC as it is for our society in general.

The evident findings in this case report are that Takotsubo cardiomyopathy has potentially lethal complications and can be caused by a media‐induced diffuse atmosphere of life threatening and panic in preconditioned patients.

## CONFLICT OF INTEREST

None declared.

## AUTHOR CONTRIBUTIONS

DH: assessed the patient, wrote the manuscript and the revision; AB: provided the MRI; SS: analyzed the ECG and implanted the sICD; all authors: involved in the clinical management of this patient and contributed to manuscript and discussion.

## ETHICAL APPROVAL

The authors fully applied the Declaration of Helsinki; and informed consent was given by the patient.
